# Unintended Effects of Cardiovascular Drugs on the Pathogenesis of Alzheimer’s Disease

**DOI:** 10.1371/journal.pone.0065232

**Published:** 2013-06-06

**Authors:** Jun Wang, Zhong Zhao, Emi Lin, Wei Zhao, Xianjuan Qian, Daniel Freire, Amanda E. Bilski, Alice Cheng, Prashant Vempati, Lap Ho, Kenjiro Ono, Masahito Yamada, Giulio M. Pasinetti

**Affiliations:** 1 Department of Neurology, Mount Sinai School of Medicine, New York, New York, United States of America; 2 Department of Neurobiology, Harvard Medical School, Boston, Massachusetts, United States of America; 3 Geriatric Research Education and Clinical Center, James J. Peters Veterans Affairs Medical Center, Bronx, New York, New York, United States of America; 4 Department of Neurology and Neurobiology and Aging, Kanazawa University Graduate School of Medical Science, Kanazawa, Japan; Boston University School of Medicine, United States of America

## Abstract

Alzheimer’s disease (AD) is rapidly becoming one of the leading causes of disability and mortality in the elderly. As life-expectancy increases, an increasing number of people will rely on modern medicines to treat age-associated disorders. Among these medications, some might benefit, while others might exacerbate, the pathogenesis of AD. We screened 1,600 FDA approved drugs for β-amyloid (Aβ)-modifying activity and identified drugs that can potentially influence amyloid precursor protein processing. In this study, we focused on cardiovascular drugs and demonstrated that some hypertensive medication can differentially modulate Aβ, both *in vitro* and *in vivo*. Our study suggests that some commonly prescribed drugs might exert unintended effects and modulate AD and provides the basis for continuing investigation of the role of individual drugs on a case-by-case basis. This line of investigation will lead to the identification of common medications that are potentially beneficial or detrimental to AD as a reference for physicians to consider when prescribing the most appropriate drugs for their patients, particularly for treating chronic disorders among the growing geriatric population.

## Introduction

Alzheimer’s disease (AD) is one of the most persistent and devastating disorders of old age, often leading to severe memory loss and functional impairment [1]. Its prevalence increases dramatically with aging. It is estimated that up to ∼5 million people in the US currently have AD and it is projected that up to 14 million people will be affected by AD by the middle of this century.

AD is characterized neuropathologically by the accumulation of extracellular neuritic plaques composed of β-amyloid (Aβ) protein, intracellular neurofibrillary tangles of hyperphosphorylated tau protein, and neuron loss [2]. A major hypothesis regarding the pathogenesis of AD is that abnormally elevated Aβ content in the brain of AD patients is critical for the development of AD dementia. This hypothesis, commonly referred to as the “amyloid hypothesis,” suggests that increasing accumulation of Aβ peptides promotes assembly of Aβ proteins into neurotoxic, extracellular soluble oligomeric Aβ aggregates that are largely responsible for cognitive deterioration and neuronal loss in AD [3]–[9]. Continuing recruitment of Aβ peptides to oligomeric Aβ aggregates leads to the formation of larger, insoluble Aβ fibrils that contribute to the formation of AD type neuritic plaques in the brain [10]. The amyloid hypothesis is supported by substantial genetic [11] and preclinical evidence [12]. However, to date, clinical trials based on amyloid hypothesis with the target mechanism of reducing brain amyloid load have produced null results including the most recently completed antibody therapies (bapineuzamab), suggesting that the amyloid hypothesis and the optimal molecular targets or the optimal timing for intervention remains to be elucidated. Nonetheless, Aβ remains to be one of the major disease modifying targets for AD drug discovery.

In addition to AD, the aged population is associated with higher risks for many chronic diseases, such as cardiovascular disorders, diabetes, arthritis, cancer or cognitive impairment. Many elderly individuals require one or more medications to treat and/or manage age-related chronic disease. A survey conducted by Boston University Epidemiology Center showed that over 80% of people 65 years and older take at least one medication and almost 50% take three or more - As life-expectancy increases with advances in medicine the number of elderly continues to increase. Thus, the incidence and prevalence of chronic disease is rising dramatically, together with the number of elderly requiring one or more medications.

We note that many drugs such as some pain medications, antihistamines and anti-psychotic medications might have adverse effect on cognition, especially in elderly. For example, a recently published prospective study involving thirteen thousand participants over 65 years of age showed that the use of anticholinergic medication increases the cumulative risk of cognitive deterioration and mortality [13]. This also raises concerns for other medications that have mild anticholinergic activities, such as cardiovascular drugs digoxin, warfarin, analgesics, codeine and prednisone. On the other hand, some medications might have positive effects on cognition. For example, recent evidence strongly supports the possibility that the use of some antihypertensive drugs such as β-blockers or Ca^++^ channel receptor antagonists, and certain potassium-sparing antihypertensive diuretics, may decrease the incidence of AD [14]–[17]. More recently, Hajjar et. al found that patients with or without AD, treated with angiotensin receptor blockers (ARBs), showed much lower amyloid burden compared to patients treated with other anti-hypertensisive medication [18].

Despite evidence associating some drugs with unintended activities on cognitive dysfunction, we do not know the potential role of how specific medications promote or inhibit the generation of Aβ.

Since Aβ is one of the major contributory factors responsible for AD-type dementia, we surveyed 1600 FDA approved drugs that are commonly used by the general population for their ability to modulate Aβ accumulation. Our intention is to identify medications that might be potentially beneficial or harmful for Aβ-mediated cognitive dysfunction. Outcomes from our studies will provide important information for physicians to consider when making decisions on prescribing the most appropriate drugs for their patients, particularly for treating chronic disorders among the growing geriatric population. Moreover, our observations also provide the impetus for future studies to systematically evaluate FDA- approved medications for potential repurposing as novel reagents to prevent or treat AD dementia.

## Materials and Methods

### Drug Screening Procedure

Embryonic-day (E)16 cortico-hippocampal neuronal cultures were prepared from heterozygous Tg2576 transgenic mice (Tg2576 neurons) [19]; [20]. Neurons were seeded onto poly-D-lysine–coated 96-well plates at 1.0×10^5^ cells per well and cultured in Neurobasal medium supplemented with 2% B27, 0.5 mM L-glutamine and 1% penicillin-streptomycin (Gibco-BRL) in the tissue culture incubator at 37°C with 5% CO_2_. The absence of astrocytes (<2%) was confirmed by the virtual absence of glial fibrillary acidic (GFAP) protein immunostaining (data not shown). For primary screening, cultured neurons were treated with 100 µM of drug in duplicates; all drugs were obtained in stock from MicroSource Discovery Systems Inc (Gaylordsville, CT). Conditioned medium was collected for Aβ detection. For secondary screening, primary neurons prepared in 96-well plates were treated with 0.1 µM, 1 µM, 10 µM, 50 µM, and 100 µM of each drug in duplicate for ∼16 hours and conditioned medium was tested for Aβ content. Cell viability was assessed using a commercial available LDH assay kit according to the manufacture’s instruction (Promega) and by MTT (3-(4,5-Dimethylthiazol-2-yl)-2,5-diphenyltetrazolium bromide) assay.

### Animals and Treatment

Female Tg2576 mice transgenic mice carrying a human amyloid precursor protein (APP) containing the familial Swedish KM670/671NL double mutation [21] were purchased from Taconic. Tg2576 mouse is a well characterized rodent of model with Aβ-mediated neuropathology and cognitive impairment and were previously used in our studies on valsartan and carvedilol [20]; [22].

Testing drugs were prepared to desired concentrations and delivered to mice by diluting them directly into the drinking water. Typically, a 25∼30 g mouse drinks ∼4–5 ml per day. The amount of testing drugs to be diluted into the drinking water was based on 1) average daily water consumption and 2) the targeted dose of testing drugs to be delivered to animals. For example, a 25 g mouse would need to take 0.375 mg propranolol per day to achieve 15 mg/kg body weight/day as in the instance of chronic propranolol treatment. Typically a 25 g mouse drinks 4.5 ml liquid per day, therefore, propranolol was diluted in the drinking water to a concentration of 0.083 mg/ml or 83 mg/L. The average liquid consumption was monitored and doses were adjusted accordingly. We acknowledge that there is variability in the amount of liquid each animal consumed. Animals under treatment were provided *ad libitum* access to testing drug-infused water as the sole source of drinking fluid.

For short-term treatment, Tg2576 mice were treated with individual compounds starting at 6 months of age and the treatment lasted one month.

For chronic treatment, Tg2576 mice were treated with 17.2 mg/kg/day nicardipine (equivalent to human 100 mg/day) or 15 mg/kg/day propranolol (equivalent to human 90 mg/day) delivered through their drinking water for 6 months, starting at 8 months of age.

All mice were housed with food *ad libitum* and maintained on a 12∶12-h light/dark cycle with lights on at 07∶00 h in a temperature-controlled (20±2°C) room prior to experimental manipulation. Mice were group housed with 3–5 mice per cage and average food and water intake was measured weekly. All procedures and protocols were approved by the Mount Sinai School of Medicine’s Institutional Animal Care and Use Committee (IACUC) through the Center for Comparative Medicine and Surgery.

### Blood Pressure Measurements

Blood pressure and heart rate were measured using a non-invasive commercial blood pressure analysis system designed specifically for small rodents (Hatteras Instruments, NC) as previously described [20]; [23]. Mice were temporarily immobilized in a restraining chamber with the tail inserted through the tail cuff, laid down into the tail slot and secured with a piece of tape. Every mouse underwent 5 preliminary cycles for acclamation and the following 10 measurements of systolic, diastolic, and mean arterial pressure (MAP) and heart rate was recorded.

### Behavioral Assessment of Cognitive Functions by the Morris Water Maze (MWM) Test

Spatial learning memory was assessed by the Morris water maze behavioral test, as previously described [20]; [24]. Briefly, mice were tested in a circular pool filled with water mixed with non-toxic white paint (Dick Blick Art Materials, IL). The water temperature was kept between 70 and 74 °F. Mice were first tested in a visible trial for 3 consecutive days where the escape platform was clearly marked with a white sail. Following the visible trial, the white sail was removed and replaced by local visual cues. Mice were trained to mount the submerged escape platform in a restricted region of the pool using the visual cues. Mice were given 4 trials per day with 60 seconds per trial. Each day, the mice would start at different quadrant and if the testing mouse failed to reach the platform in 60 seconds, it would be gently led to the platform and let stay on the platform for 15 seconds before returning to the home cage. Spatial memory was assessed by recording the latency time for the animal to escape from the water onto a submerged escape platform as a function of the number of learning trials during the learning phase. Twenty-four hours after the last learning session, mice were subjected to a 45 second probe trial wherein the escape platform is removed. The water maze activity was monitored with the San Diego Instrument Poly-Track video tracking system (San Diego, CA). The cued-platform learning curve was used as control for the non-spatial factors on MWM performance, e.g., sensory-motor performance, motivation, anxiety etc., which can be influenced by the potential effect of the testing drug. The mentstrual cycle was not controlled for the behavior testing.

### Assessment of AD-type Amyloid Neuropathology

Total Aβ1-40 or Aβ1-42 in the brain and in plasma were quantified by sandwich ELISA, as previously described [25]. Specifically, frozen pulverized tissue was homogenized in 5.0 M guanidine buffer, diluted (1∶10) in phosphate-buffered saline containing 0.05% (v/v) Tween-20 and 1 mM Pefabloc protease inhibitors (Roche Biochemicals, Indianapolis, IN) and centrifuged for 20 min at 4°C. Supernatant was subjected to Aβ_1–40_ or Aβ_1–42_ quantification by sandwich ELISA (BioSource, Camarillo, CA).

### Statistical Analysis

Differences between means were analyzed using two-tailed Student t-test. For behavior testing, data were analyzed using two-way repeated measures ANOVA followed by Newman-Keuls post-hoc analysis. In all analyses, the null hypothesis was rejected at the 0.05 level. All values are expressed as mean and standard error of the mean (SEM). All statistical analyses were performed using the prism Stat program (GraphPad Software, Inc.).

## Results

### Identification of Cardiovascular Drugs with AD-modifying Activity

**Figure 1:**
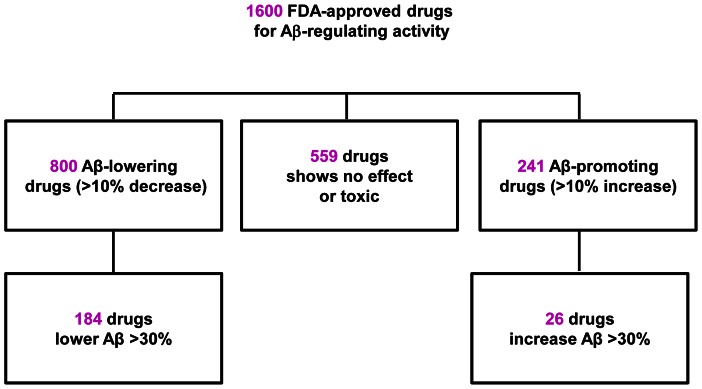
Schematic diagram of primary screening of 1600 FDA approved drugs.

Our high throughput screening study assessed 1600 FDA approved drugs for their ability to modulate Aβ activity. We found 559 drugs of the 1600 had no effect on APP processing or were toxic to neurons at the testing concentration, while 800 drugs could reduce Aβ content over 10% in primary neurons derived from Tg2576 mice, among which, 184 drugs were able to reduce Aβ content greater than 30% compared to vehicle. We also found 241 drugs could potentially promote Aβ accumulation including 26 drugs that could increase the level of Aβ greater than 30% compared to vehicle treatment (Figure 1).

Cardiovascular medications are one of the most commonly prescribed drugs, especially in the elderly. Among the 1600 drugs we tested, 115 are cardiovascular drugs representing all pharmacological classes of antihypertensive, antithrombotic and fibrinolytic, antianginal, antiarrhythmic and congestive heart failure medications (Table 1). Primary screening of these drugs identified 13 cardiovascular drugs that could reduce ≥30% Aβ accumulation in the conditioning medium from Tg2576 neurons following 16 hours treatment at 100 µM concentration without toxicity as tested by MTT assay and LDH release assay (Table 1). We continued our secondary screening for dose response on these candidates and found that among the 13 candidates, carvedilol, propranolol, valsartan, losartan, hydralazine, nicardipine and amiloride demonstrated a concentration-dependent reduction of Aβ_1–40_ and Aβ_1–42_ in primary embryonic cortico-hippocampal neuron cultures. All 7 drugs are antihypertensive agents of different pharmacological subclasses. We also found that 2 drugs, namely trandolapril and furosemide, could promote ≥30% Aβ accumulation compared to vehicle control.

**Table 1:** 115 commonly prescribed cardiovascular drugs used in screening for potential Aβ-modifying activity.

Drug Name	Aβ		Drug Name	Aβ	Drug Name		Aβ
**ANTIHYPERTENSIVE**							
**A. β-Adrenergic blocker**			**D. Vasodilator**		**G. Angiotensin converting enzyme inhibitor**		
RESERPINE	**–**		PAPAVERINE HYDROCHLORIDE	**–**	PERINDOPRIL ERBUMINE	83.8	
**CARVEDILOL**	**38.3**		**HYDRALAZINE**	**66.1**	FOSINOPRIL SODIUM	94.7	
**PROPRANOLOL HYDROCHLORIDE (−)**	**55.0**		DIPYRIDAMOLE	72.2	ENALAPRIL MALEATE	100.7	
**PROPRANOLOL HYDROCHLORIDE**	**65.2**		ISOXSUPRINE HYDROCHLORIDE	78.3	CAPTOPRIL	104.5	
NYLIDRIN HDROCHLORIDE	70.5		NICOTINYL TARTRATE	80.0	RAMIPRIL	107.4	
LABETALOL HYDROCHLORIDE	84.9		ISOSORBIDE DINITRATE	80.5	BENAZEPRIL HYDROCHLORIDE	116.3	
ATENOLOL	90.4		PROTOVERATRINE A	91.7	QUINAPRIL HYDROCHLORIDE	119.3	
METOPROLOL TARTRATE	91.3		MINOXIDIL	94.2	***TRANDOLAPRIL***	***136.7***	
ALPRENOLOL	93.8		VINCAMINE	99.1	**H. Diuretic**		
NADOLOL	94.2		METHYLDOPA	104.0	BUMETANIDE		–
PINDOLOL	99.0		MOLSIDOMINE	118.3	ETHACRYNIC ACID		–
PRACTOLOL	99.7		DIAZOXIDE	113.5	TRIAMTERENE		–
TIMOLOL MALEATE	101.3		ADENOSINE PHOSPHATE	121.8	**AMILORIDE**		**60.4**
GUANETHIDINE SULFATE	125.5		**E. Ganglionic blocking agent**		CYCLOTHIAZIDE		71.4
ACEBUTOLOL HYDROCHLORIDE	133.6		PEMPIDINE TARTRATE	80.9	ALTHIAZIDE		74.4
**B. a-Adrenergic Blocker**			HEXAMETHONIUM BROMIDE	84.8	BENDROFUMETHIAZIDE		76.2
PRAZOSIN HYDROCHLORIDE	–		PENTOLINIUM TARTRATE	121.0	SPIRONOLACTONE		77.5
PHENTOLAMINE HYDROCHLORIDE	83.4		MECAMYLAMINE HYDROCHLORIDE	124.4	TRICHLORMETHIAZIDE		89.3
PHENOXYBENZAMINE HYDROCHLORIDE	87.5		**F. Ca channel blocker**		HYDROCHLOROTHIAZIDE		89.5
TOLAZOLINE HYDROCHLORIDE	90.8		AMLODIPINE BESYLATE	–	METOLAZONE		91.4
URAPIDIL	96.4		BEPRIDIL HYDROCHLORIDE	–	CLOPAMIDE		92.0
TAMSULOSIN HYDROCHLORIDE	106.5		FENDILINE HYDROCHLORIDE	–	HYDROFLUMETHIAZIDE		92.4
GUANABENZ ACETATE	131.2		FLUNARIZINE HYDROCHLORIDE	–	UREA		92.8
**C. Angiotensin receptor blocker**			TETRANDRINE	–	CANRENOIC ACID, POTASSIUM SALT		100.6
CANDESARTAN CILEXTIL	–		**NICARDIPINE**	**10.7**	THEOBROMINE		101.3
**VALSARTAN**	**51.6**		NITRENDIPINE	33.0	BENZTHIAZIDE		105.7
**LOSARTAN**	**70.0**		VERAPAMIL HYDROCHLORIDE	70.9	INDAPAMIDE		107.0
OLMESARTAN CILEXTIL	83.9		NIMODIPINE	79.1	CHLOROTHIAZIDE		107.3
IRBESARTAN	89.7		NIFEDIPINE	83.4	TORSEMIDE		107.4
TELMISARTAN	91.1		DILTIAZEM HYDROCHLORIDE	107.8	CHLORTHALIDONE		108.5
			BERBAMINE HYDROCHLORIDE	119.1	***FUROSEMIDE***		***138.5***
**ANTIARRHYTHMIC**							
PROPAFENONE HYDROCHLORIDE	–		DISOPYRAMIDE PHOSPHATE	83.5	AJMALINE	114.0	
AMIODARONE HYDROCHLORIDE	–		MEXILETINE HYDROCHLORIDE	88.8	PROCAINAMIDE HYDROCHLORIDE	118.1	
QUINIDINE GLUCONATE	63.3		HYDROQUINIDINE	96.5			
**ANTITHROMBOTIC and FIBRINOLYTIC**							
**A. Coagulant**			**B. Antifibrinolytic**		**C. Antihyperlipidemic**		
SULOCTIDIL	–		TRANEXAMIC ACID	84.9	FENOFIBRATE	–	
DICUMAROL	51.3		AMINOCAPROIC ACID	124.4	SIMVASTATIN	–	
SCOPOLETIN	71.9				ATORVASTATIN CALCIUM	66.0	
WARFARIN	89.4				ROSUVASTATIN	83.6	
ANISINDIONE	90.1				PROBUCOL	86.8	
PENTOXIFYLLINE	97.1				CLOFIBRATE	90.9	
PHENINDIONE	104.0				NIACIN	101.1	
					BEZAFIBRATE	107.2	
**CONGESTIVE HEART FAILURE**							
LANATOSIDE C		–	DOPAMINE HYDROCHLORIDE	85.0	PERUVOSIDE	115.3	
DIGITOXIN		68.3	DOBUTAMINE HYDROCHLORIDE	90.1			

The 115 cardiovascular agents were obtained as part of the Spectrum Collection from MicroSource Discovery Systems Inc. and listed in 4 pharmacological categories (source: Physician’s Desk Reference and Martindale Complete Drug Reference). Potential Aβ-modifying activity was assessed using primary cortico-hippocampal neuron cultures derived from embryonic Tg2576 AD mice and the levels of Aβ in the conditioned medium were measured and expressed as percentage of vehicle-treated control (Aβ column). In the high-throughput screening studies, 13 drugs lowered the Aβ content greater than 30% and two drugs (in bold and Italic) increased Aβ content greater than 30% compared to the vehicle controls. The 13 Aβ-lowering drugs were proceeded to a follow-up dose dependent test and the 8 drugs in bold were found to exert significant dose dependent Aβ -lowering activity in the absence of cellular toxicity. “–” indicates that the drug is toxic for primary neurons at 100 µM.

### Short-term *in vivo* Efficacy Study using Tg2576 Mouse Model of AD

Based on the *in vitro* dose-dependent results, we tested 8 drugs (6 with Aβ-lowering activity and 2 with Aβ-promoting activity, Table 2) to explore whether these drugs can exert similar Aβ modification activity in an experimental model of AD.

**Table 2:** Short-term in vivo treatment dosage conversion in Tg2576 mice.

		Equivalent human dose	Recommended dose for
	Tg2576 treatment	Prescribed clinical dose	hypertension treatment in human
	(mg/kg/day)	(mg/day)	(mg/day)
**propranolol**	41.3	240	120∼240
**carvedilol**	8.6	50	25∼50
**nicardipine**	17	100	50∼100
**losartan**	20.6	120	60∼120
**amiloride**	3.4	20	5∼20
**hydralazine**	51.7	300	100∼300
**furosemide**	14.8	80	20∼80
**trandolopril**	0.74	2	1∼2

The dosage recommended for hypertension treatment in humans is listed as prescribed clinical dose. The equivalent dosage in animals is calculated using FDA criteria for converting drug equivalent dosages across species, based on body surface area [39]. The mice equivalent dosage was used in the short-term treatment.

We performed a short-term feasibility study using the Tg2576 mice model of AD. We used the doses equivalent to the recommended dose for treating cardiovascular disease in humans (Table 2). All dosage ranges were obtained from online Physicians’ Desk Reference (http://www.pdr.net) and the conversions of drug equivalent dosages across species were derived using FDA criteria based on body surface area [26]. The detailed dosage for the treatment is listed in Table 2.

**Figure 2:**
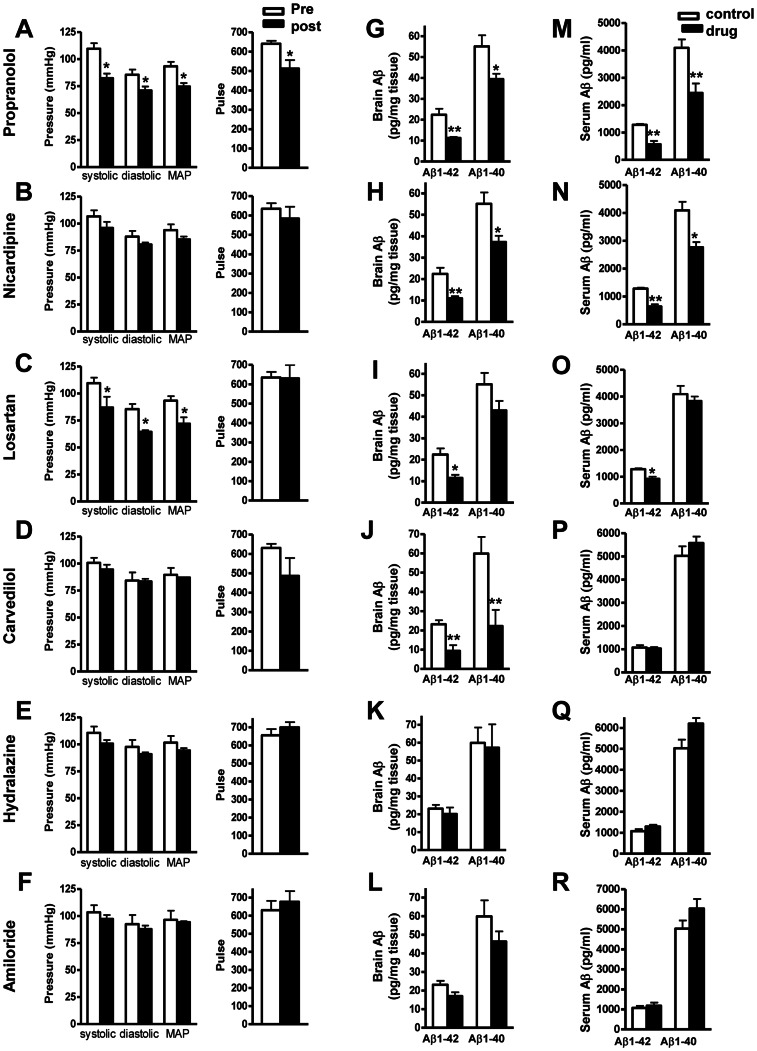
Effect of Aβ-lowering drug treatment on blood pressure and amyloid neuropathology in Tg2576 mice. (A–F) Measurements of systolic, diastolic blood pressure, and mean arterial blood pressure (MAP) and heart beat (pulse) in response to ∼4 weeks of drug treatments. (G-L) Assessment of Aβ1-42 and Aβ1-40 peptide concentrations in the brain of drug treated mice *vs.* the control mice. (M-R) Assessment of Aβ1-42 and Aβ1-40 in peripheral blood of drug treated mice *vs.* the control mice. Blood pressure determination for each animal was calculated as the mean of 10 individual measurements. Values represents group mean values (+SEM); n = 3–5 mice per group. **P<0.01, *P<0.05, 2-tailed student t-test.

We found that short-term drug treatment for one month in Tg2576 mice, delivered in the drinking water at clinical dosage, did not significantly influence animal body weight, except for hydralazine treatment (Table 3), which showed a significant body weight drop following 1 month treatment. However, propranolol and losartan treatment resulted in a significantly influenced blood pressure, ∼20% drop in systolic, diastolic and mean arterial pressure (MAP) measurements was observed, while other drugs showed no effect on the blood pressure measurements in the normotensive mice (Figure 2, A-F). The lack of hypotensive effect of the other drugs can be due to: 1) some of the drugs, such as nicardipine, significantly reduce the blood pressure in hypertensive subjects, but have minimal effect on blood pressure in normotensive subjects. 2) the drug dose conversion between species we used in the study is based on the body surface area. It is possible that the absorption and metabolism of certain drug might be different in mice compared to humans.

**Table 3:** Effect of short-term in vivo on body weight and liquid consumption in Tg2576 mice.

Drug	Body Weight (g)		Liquid Consumption (ml/day)
	Pretreatment	Posttreatment	
Propranolol	23.2±1.0	19.9±2.6	4.8±0.3
Nicardipine	21.9±1.9	19.7±1.6	4.8±0.2
Losartan	24.7±2.6	23.7±2.0	4.6±0.5
Carvedilol	27.4±1.8	27.3±1.5	4.3±0.5
Hydralazine	27.6±1.0	24.3±1.0*	4.5±0.5
Amiloride	30.3±8.5	27.4±9.8	4.8±0.3
Trandolapril	32.9±13.6	32.9±12.7	4.5±0.8
Furosemide	34.5±3.8	32.2±6.8	4.9±0.6

Animals were treated with the anti-hypertensive drugs for four weeks and body weight and liquid consumption were monitored weekly. Data presented here are the end point body weight and the average liquid consumption throughout the study.

* P<0.05, 2-tailed student t-test, n = 3–5 for each treatment group.

Consistent with the in vitro data, treatment of Tg2576 mice with propranolol, nicardipine or carvedilol resulted in a ∼40% reduction in total guanidine-extractable Aβ1-42 peptide and Aβ1-40 peptide in the brain (Figure 2G, 2H, and 2J), while treatment with hydralazine and amiloride had no effect on brain amyloid peptides levels. Losartan treated mice showed a significant reduction of Aβ1-42 in the brain while no change in Aβ1-40 level. We also found that propranolol, nicardipine and losartan treatment resulted in significant reductions of plasma level of Aβ (Fig 2M, 2N and 2O).

**Figure 3:**
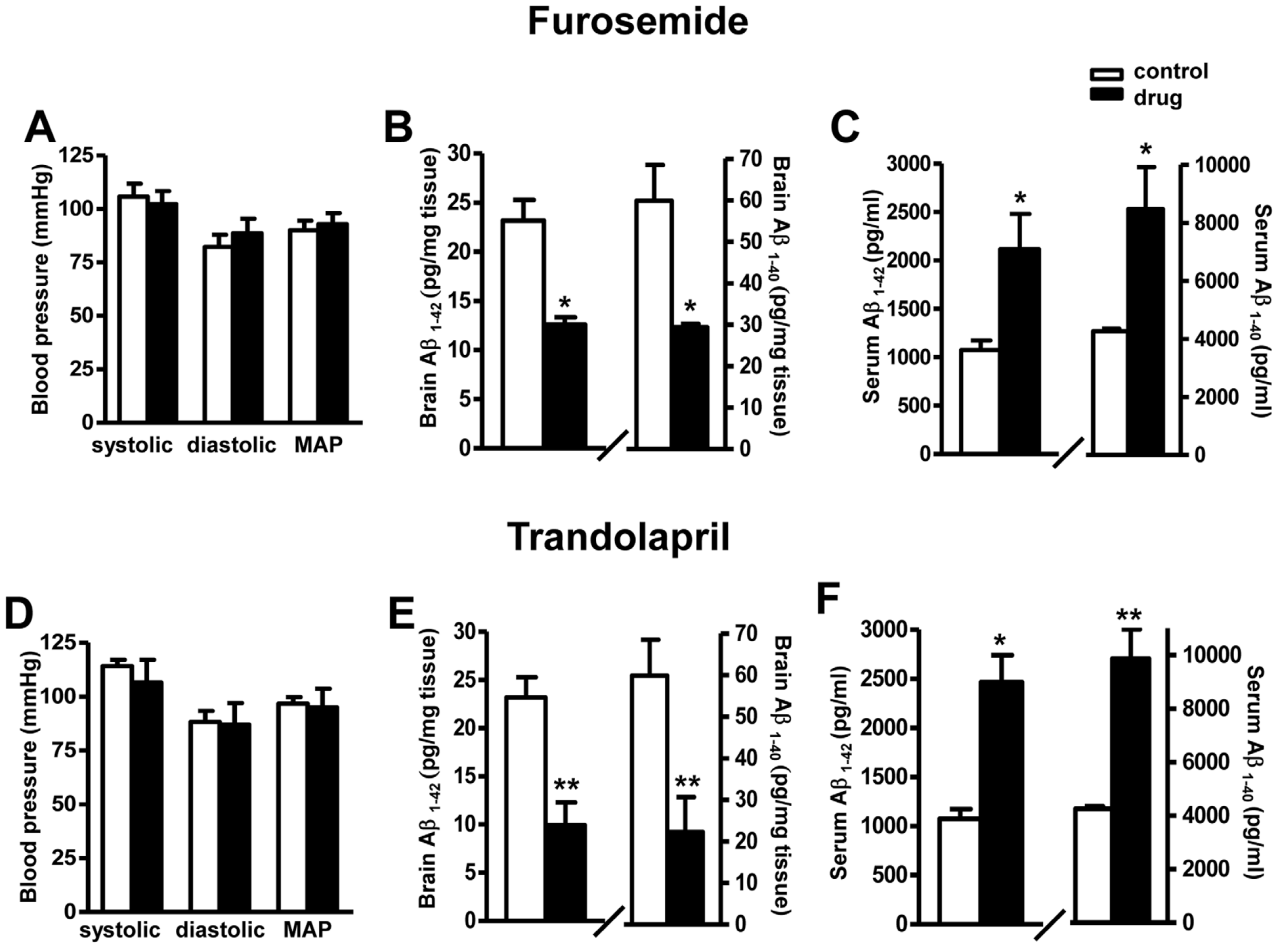
Effect of Aβ -promoting drugs treatment on blood pressure and amyloid neuropathology in Tg2576 mice. (A and D) Measurements of systolic, diastolic blood pressure, and mean arterial blood pressure (MAP) in response to ∼4 weeks of drug treatments. (B and E) Assessment of Aβ1-42 and Aβ1-40 peptide concentrations in the brain of drug treated mice *vs.* the control mice. (C and F) Assessment of Aβ1-42 and Aβ1-40 in peripheral blood of drug treated mice *vs.* the control mice. Values represents group mean values (±SEM); n = 3–5 mice per group. **P<0.01, *P<0.05, 2-tailed student t-test.

To our surprise, the drugs that increased Aβ *in vitro*, at the dosage equivalent to human prescription dosage (14.8 mg/kg/day for furosemide and 0.74 mg/kg/day for trandolapril), did not increase total Aβ1-40 or Aβ1-42 in the brain following one-month treatment. On the contrary, both furosemide and trandolapril treatment significantly reduced the levels of total brain amyloid content in the Tg2576 mice following one-months short-term treatment (Figure 3B and 3E) without significantly changing of blood pressure. The reduction of brain Aβ content in both treatments was associated with significant increases of plasma levels of Aβ (Figure 3C and 3F). In parallel studies, we confirmed that the drug treatments did not alter the expression of APP in the brains of the mice using western blot analysis (data not shown).

### Chronic Administration of Nicardipine and Propranolol on Cognitive Function and Brain Neuropathology

The validation of the effect of any medication on AD pathology is cognitive function.

Based on the results from the short-term study (Figure 2), we chose nicardipine and propranolol, both of which significantly changed the levels of total Aβ1-40 and Aβ1-42 in the brain following one-month treatment for chronic studies, to evaluate their effect on cognitive function. Since short-term treatment with propranolol resulted in a significant hypotensive effect in the normatensive mice and significantly reduced heart rate (Figure 2A), we adjusted the dose to 15 mg/kg/day for chronic studies. We used the same dose for nicardipine as short-term treatment since it did not have any adverse effect.

**Figure 4:**
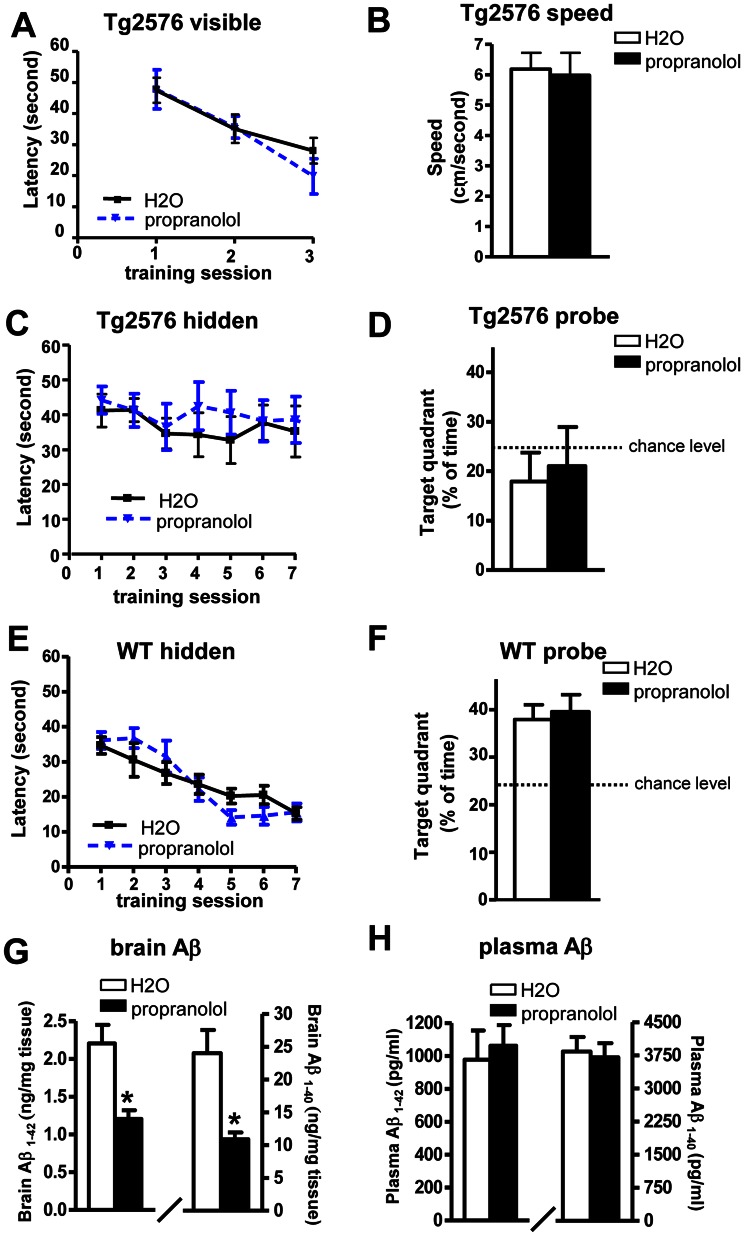
Effect of chronic propranolol treatment on spatial memory and neuropathology in Tg2576 mice. (A-D) The influence of chronic propranolol treatment on Aβ related spatial memory in Tg2576 mice by Morris water maze test (A) Visible trial (B) Swimming speed (C) Hidden platform learning acquisition, latency score represents time taken to escape to the platform from the water. (D) Probe trial. Percent of time in quadrant is calculated as the ratio of time spent in the target quadrant area relative to the time spent in the rest of the pool. (E-F) The influence of chronic propranolol treatment wild type mice by Morris water maze test: (E) Hidden platform learning acquisition (F) Probe trial. (G-H) Brain and plasma levels of total Aβ1-40 and Aβ1-42 peptides following chronic propranolol treatment. Values represent group mean ± SEM, n = 7–8 per group.

Following 6 months treatment, we tested spatial memory function in the Tg2576 mice using the Morris water maze test. First, we used the visible trial to confirm that propranolol treatment did not affect any non-spatial factors e.g., sensory-motor performance, motivation, anxiety etc. which might affect their water maze performance. Both groups were able to identify the target platform and both groups had similar swimming speed (Figure 4A and 4B). In the hidden platform training session, we found that 6 months of chronic propranolol treatment did not affect AD-type cognitive deterioration reflected by equally impaired spatial memory function between the treated and non-treated control Tg2576 mice. Neither group showed significant learning during the 7 day hidden platform testing (Figure 4C). This lack of learning was also evident during the probe trial, as neither group spent more than 25% chance time in the target quadrant (Figure 4D). Since propranolol is a non-selective beta adrenergic receptor blocker that has been used for memory relief [27]–[29], in parallel studies, we tested whether it will interfere with spatial memory in wild type (WT) mice. WT mice were treated with the same treatment regime for 6 months and subjected to MWM test. We found propranolol at 15 mg/kg/day dose did not affect spatial memory in WT mice, as reflected by their normal learning progress during the 7 day training (Figure 4E). The probe trial also confirms that both propranolol-treated and non-treated control WT mice spent significantly more time in the target quadrant (∼40% of time, much more than the 25% chance level, Figure 4F), indicating that propranolol at the treatment dose did not affect spatial memory function.

We next measured the brain neuropathology and found that, similar to the short-term treatment, 6 months of chronic propranolol treatment significantly reduced the level of both Aβ1-40 and Aβ1-42 in the brains compared to the control non-treated mice (Figure 4G). However, measurements of plasma levels of Aβ showed that there was no change in Aβ1-40 and Aβ1-42 (Figure 4H) which is different from the short-term treatment results (Figure 2M).

**Figure 5:**
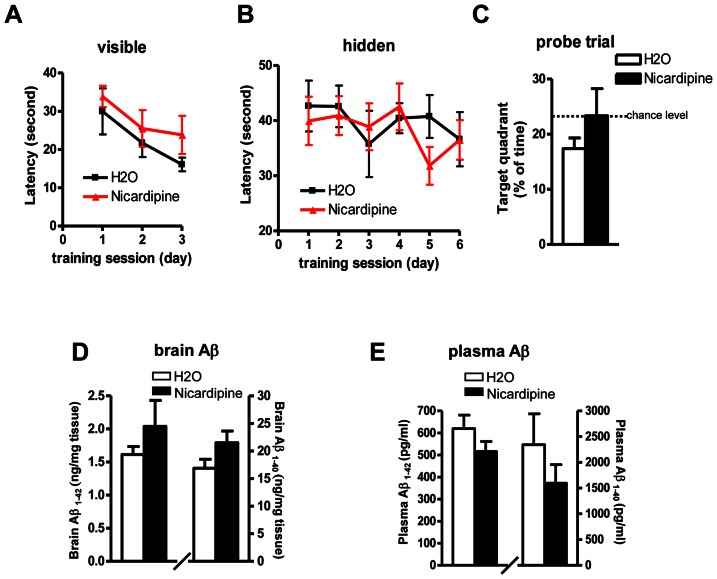
Effect of chronic nicardipine treatment on spatial memory and neuropathology in Tg2576 mice. (A-C) The influence of chronic nicardipine treatment on Aβ related spatial memory in Tg2576 mice by MWM test: (A) Visible trial (B) Hidden platform learning acquisition (C) Probe trial. (D-E) Brain and plasma levels of total Aβ1-40 and Aβ1-42 peptides following chronic nicardipine treatment. Values represent group mean ± SEM, n = 9–11 per group.

Chronic nicardipine treatment in Tg2576 showed similar behavior results. No benefits of cognitive improvement were observed following 6 months treatment by MWM test (Figures 5A–5C). Surprisingly, contrary to the short-term results that nicardipine treatment significantly lowered the brain levels of Aβ1-40 and Aβ1-42 (Figure 2H), chronic nicardipine treatment did not affect the levels of Aβ in the brain (Figure 5D). There was no difference in plasma levels of Aβ1-40 and Aβ1-42 following chronic treatment, which is also different from the short-term result that there was a significant reduction in plasma Aβ following 1 month treatment (Figure 2N).

## Discussion

AD is rapidly becoming one of the leading causes of disability and mortality, and it is expected that the prevalence of AD in the US will quadruple over the next 50 years [30]; [31]. With baby boomers reaching retirement age and life expectancy continuing to increase, an increasing number of elderly people will be taking one or more medications. Whether current commonly prescribed medications can worsen or improve AD dementia, and to what extent they might influence dementia from normal aging or from other neurodegenerative conditions, are issues with enormous public health implications.

In this study, we surveyed 1600 FDA approved drugs for their ability to modulate aberrant generation of AD-type Aβ peptides from the amyloid precursor protein (APP) process in primary cortico-hippocampal cell cultures generated from the Tg2576 mouse AD model. We found a subset of medication that significantly increased and a second subset of medications that significantly reduced generation of Aβ peptides in primary neuron cultures.

Since cardiovascular disorder is one of the most prevalent diseases among the elderly, we focused on the potential role of cardiovascular drugs in regulating Aβ generation. We found that most of the FDA-approved cardiovascular drugs have no detectable activity with regard to the generation of Aβ peptides from our *in vitro* primary cortico-hippocampal screening system. However, we identified select cardiovascular drugs, such as propranolol and nicardipine, that exert *in vitro* Aβ-lowering activity, and other cardiovascular drugs, such as furosemide, that significantly promote the generation of Aβ peptides *in vitro*.

Among the subset of cardiovascular drugs we found to promote Aβ generation *in vitro*, several (e.g., trandolapril, quinapril) are angiotensin converting enzymes (ACE) inhibitors. This observation is consistent with recent evidence suggesting ACE may be involved in the degradation of Aβ peptides [32]; [33]. Indeed, the ACE inhibitor, captopril, has previously been shown to increase Aβ accumulation *in vitro* [32]; [33]. However, in our primary screening, we found captopril barely increased Aβ levels in the conditioned medium of primary neurons following treatment. This discrepancy might have been caused by the usage of different in vitro systems. Zou et. al used COS cells for their study [33] and Hemming et. al. used CHO and HEK293 cells [32]. In both studies, ACE was over expressed in the cell lines. While in our study, we used primary neurons derived from Tg2576 mice and the inhibition was towards endogenous ACE. It is possible that ACE is not a major contributor in Aβ catabolism in primary neurons *in vitro.* Another observation is that the majority of ACE inhibiting cardiovascular drugs we screened (5 of 8 ACE inhibitors screened) did not exhibit Aβ-modification activity. Thus it is also likely that not all ACE inhibitors are equally effective in preventing Aβ degradation.

Based on evidence that trandolapril is capable of promoting Aβ generation *in vitro*, we explored the physiological impact of trandolapril on Aβ neuropathology *in vivo*. In contrast to our *in vitro* evidence, results from our short-term study in Tg2576 mice treated for 1 month with trandolapril at a dose equivalent to the clinical dosage showed that trandolapril significantly reduced Aβ contents in the brain while it significantly increased Aβ contents in the plasma. Interestingly, the other drug furosemide, which showed Aβ promoting activity *in vitro*, also reduced brain levels of Aβ accompanied by increased levels of Aβ in plasma following short-term treatment in Tg2576 mice. Based on these observations, we suggest that some of the cardiovascular drug, when administered in short-term, might promote generation of Aβ in the brain. However, the treatment might temporarily alter the dynamic balance of Aβ between brain and plasma, e.g. increase the Aβ efflux or reduce the Aβ influx which may result in the promotion of transport of Aβ from the brain to the periphery. It would be interesting to measure the levels of transport proteins such as receptor for advanced glycation end products (RAGE) and Lipoprotein receptor-related protein (LRP) following the short-term treatments.

Since most cardiovascular diseases are chronic conditions, the medications are generally prescribed for a prolonged period of time. Therefore, in our study, we also tested the effect of chronic drug application in cognitive function and brain neuropathology. Previously, we have shown that valsartan, one of the angiotensin receptor blocker medications identified in the secondary screening (Table 1), could reduce Aβ neuropathology and improve spatial memory function in Tg2576 mice following chronic treatment [20]. We also showed in another study that carvedilol, a nonselective β-adrenergic receptor blocker, can prevent cognitive deterioration and reduce brain neuropathology by interfering with Aβ oligomerization and improving basal synaptic transmission in the TgCRND8 mouse model of AD [22]; [34].

In this study, we found that chronic treatment with propranolol could significantly reduce brain amyloid neuropathology but had no effect on cognitive function. It is possible that reducing amyloid alone might not be sufficient to improve cognition. It is also possible that propranolol is able to reduce total amyloid but is not able to influence the level of soluble oligomeric Aβ species, which are increasingly regarded as neurotoxic and largely responsible for synaptic failure in AD models [6]–[8]; [35]–[37]. Brain neuropathology results from nicardipine, again suggesting that short-term treatment might temporarily alter the hemodynamic between the brain and blood resulting in increased clearance while prolong treatment may not have any effects. Future studies will focus on mechanistic investigation of how certain drugs might influence APP processing or Aβ catabolism and their possible role in Aβ oligomerization which may explain the lack of behavior improvements in propranolol treated mice.

It is not surprising that *in vitro* and *in vivo* studies showed differences in biological efficacies. First of all, brain bioavailability is one of the major obstacles and some of the drugs might not be able to pass the blood brain barrier to influence brain amyloid processing and, therefore, are not able to exert their activities in the brain. Secondly, some of the drugs might have gone through extensive metabolism and the metabolites might not behave as the original drugs. Thirdly, some of the drugs might also impact other systems (such as vessel permeability, periphery protein degradation pathway, etc.) that will eventually affect the net outcome of the drug treatment. More importantly, drugs modifying amyloid may or may not have significant impacts in modulating AD-type cognitive function.

Therefore, in order to identify drugs that might have beneficial effects, such as reducing Aβ neuropathology and improving cognitive function, for the consideration of physicians over drugs that might be potentially detrimental to cognition, individual drugs must be investigated on a case by case basis and these results should be supplemented with data obtained from clinical studies with patients following long-term drug treatment. A good example is that our preclinical study demonstrated that chronic application of ARB valsartan can significantly reduce the levels of soluble Aβ as well as total amyloid plaque load in the brain in a mouse model of AD [20]. Data collected from the National Alzheimer Coordinating Center (including 29 centers across the US) confirms that patients receiving ARB medication have less amyloid deposition compared to untreated patients or patients with non-ARB antihypertensive medications [38]. Collectively, these studies may provide useful information for physicians when prescribing antihypertensive drugs.
